# Non-physical practice improves task performance in an unstable, perturbed environment: motor imagery and observational balance training

**DOI:** 10.3389/fnhum.2014.00972

**Published:** 2014-12-04

**Authors:** Wolfgang Taube, Michael Lorch, Sibylle Zeiter, Martin Keller

**Affiliations:** ^1^Department of Medicine, Movement and Sport Science, University of FribourgFribourg, Switzerland; ^2^Department of Sport Science, University of FreiburgFreiburg, Germany; ^3^Department of Medicine, Human Movement and Sport Sciences, Universities of Geneva and LausanneGeneva, Switzerland

**Keywords:** mental training, motor imagery learning, observational learning, balance control, posture control

## Abstract

For consciously performed motor tasks executed in a defined and constant way, both motor imagery (MI) and action observation (AO) have been shown to promote motor learning. It is not known whether these forms of non-physical training also improve motor actions when these actions have to be variably applied in an unstable and unpredictable environment. The present study therefore investigated the influence of MI balance training (MI_BT) and a balance training combining AO and MI (AO+MI_BT) on postural control of undisturbed and disturbed upright stance on unstable ground. As spinal reflex excitability after classical (i.e., physical) balance training (BT) is generally decreased, we tested whether non-physical BT also has an impact on spinal reflex circuits. Thirty-six participants were randomly allocated into an MI_BT group, in which participants imagined postural exercises, an AO+MI_BT group, in which participants observed videos of other people performing balance exercises and imagined being the person in the video, and a non-active control group (CON). Before and after 4 weeks of non-physical training, balance performance was assessed on a free-moving platform during stance without perturbation and during perturbed stance. Soleus H-reflexes were recorded during stable and unstable stance. The post-measurement revealed significantly decreased postural sway during undisturbed and disturbed stance after both MI_BT and AO+MI_BT. Spinal reflex excitability remained unchanged. This is the first study showing that non-physical training (MI_BT and AO+MI_BT) not only promotes motor learning of “rigid” postural tasks but also improves performance of highly variable and unpredictable balance actions. These findings may be relevant to improve postural control and thus reduce the risk of falls in temporarily immobilized patients.

## Introduction

Postural control is important not only for many activities of daily living but also to succeed in locomotion and sports or simply to avoid injuries due to loss of balance. Therefore, there are various ways to promote balance performance, especially in populations that are at special risk of falls and injuries such as children, elderly people or people engaged in sports (Taube et al., 2008; Granacher et al., 2011). However, although there are great differences in the way balance skills are promoted, all conventional interventions have in common that people have to train physically. Thus, individuals suffering from immobilization due to injuries, diseases or restricted environments (e.g., space flights) do not have the opportunity to train their postural skills. As a consequence, long periods of immobilization (e.g., after hip fracture) are often followed by a fear of falling that is associated with several negative rehabilitation outcomes such as less time spent on exercise, increased rate of future falls, institutionalization, loss of mobility and increased mortality risk (Visschedijk et al., 2010). To counteract the inability to train postural control during immobilization, it has been suggested that a “non-physical kind of training” such as motor imagery of movements (Hamel and Lajoie, 2005) or observation of postural tasks (Tia et al., 2010) should be applied. The rationale for doing so is that previous studies have indicated that physical and mental training share common neural sources (Roth et al., 1996; Ehrsson et al., 2003). Similarly, it was argued that some identical neuronal networks are activated independent of whether a motor task is performed by oneself or another person is observed doing the same task (Rizzolatti, 2005). In a recent study, we compared brain activity by means of functional magnetic resonance imaging during motor imagery (MI) and action observation (AO) of differently demanding balance tasks (Taube et al., 2015). Brain activity was higher when subjects imagined the postural task (MI) compared to simply watching a video displaying the task (AO). However, when subjects were encouraged to watch the videos and imagine they were the person displayed in the videos (called “AO+MI”), even greater activities were evident in the pre-motor cortices, the SMA, the primary motor cortex, the putamen, and the cerebellum. In general, independent whether subjects observed or imagined the postural task, brain activity in SMA, cerebellum and basal ganglia was greater when the task consisted of a challenging postural task (dynamic perturbation) compared to a simple, static task (standing). Consequently, we instructed subjects to use the two most successful mental conditions in the present study, namely “AO+MI” and “MI,” in order to test whether non-physical balance training of challenging postural tasks leads to similar behavioral adaptations than conventional physical balance training. Previously, Hamel and Lajoie (2005) reported significantly decreased postural oscillations in anterior-posterior direction in upright standing elderly participants who underwent 6 weeks of mental postural training. Similarly, Tia et al. (2010) reported beneficial effects after observational training in elderly participants: namely, increased walking speed and decreased duration when sitting down after observing walking and sit-to-stand/back-to-sit tasks, respectively, during the training period. Thus, there is preliminary evidence that both motor imagery and observation of postural skills may improve those actions. However, so far only movements on stable ground have been investigated. Thus, participants did not have to counteract uneven terrain or external perturbations. In this kind of context, a postural task does not greatly differ from a fine motor skill, as the trajectory of the movement can be anticipated and mentally rehearsed in a stable and rigid way. Furthermore, easy to perform balance tasks such as standing on both feet on a force plate were shown to lack functional relevance (Schieppati et al., 1994) and are unreliable predictors for the risk of falling (Horak et al., 1992). Consequently, static balance tests are not the best indicator for functional postural stability. Thus, the question remains whether MI and AO+MI can improve performance in an unstable environment where external perturbations have to be counteracted that cannot precisely be anticipated. The current study therefore aimed to clarify the influence of MI_BT and AO+MI_BT on postural control of undisturbed and disturbed upright stance on unstable ground.

Furthermore, as conventional (physical) balance training (BT) is known to have not only a positive influence on balance skills but also on explosive strength (Gruber et al., 2007a) and jumping abilities (Taube et al., 2007b), we tested whether non-physical BT has similar effects. Physical BT leads to plasticity in cortical structures such as the primary motor cortex (Beck et al., 2007; Taube et al., 2007a; Schubert et al., 2008) and the SMA (Taubert et al., 2010) and it was previously argued that increases in explosive strength and jump height after BT may rely at least partly on adaptations of these structures (Taube et al., 2008). As MI and AO+MI of postural tasks can activate these brain areas (Taube et al., 2015) it seems at least conceivable that non-physical BT possesses similar transfer abilities than physical BT.

Based on the observation that the H-reflex was modulated during motor imagery of motor actions (Oishi et al., 1994; Hale et al., 2003) and after 10 weeks of mental up- or down-training of the H-reflex (Thompson et al., 2009), it was hypothesized that mental balance training alters spinal reflex excitability. As previous studies have indicated that several weeks of physical balance training lead to an H-reflex suppression (Taube et al., 2007a), probably by increasing the supraspinal induced pre-synaptic inhibition, a reduction of the H-reflex was expected after mental training.

## Materials and methods

### Study participants

Thirty-six healthy participants without neurological or orthopedic disorders participated in the present study and were randomly allocated to one of three groups: (a) observational balance training group (AO+MI_BT; *n* = 12; 25 ± 4 years; 177 ± 6 cm; 73 ± 12 kg; 7 females), (b) motor imagery balance training group (MI_BT; *n* = 12; 23 ± 3 years; 174 ± 4 cm; 68 ± 7 kg; 8 females; one participant was excluded from the post-measurement due to an insufficient number of training sessions) or (c) control group (CON; *n* = 12; 24 ± 4 years; 171 ± 6 cm; 66 ± 7 kg; 7 females). None of the participants participated in any other systematic training during the experiment or had previously performed any mental training or conventional (physical) balance training. Before testing, all participants were informed about the experiments and gave written informed consent for the experimental procedure. The study was approved by the local ethics committee of Fribourg and is in accordance with the Declaration of Helsinki.

### Training interventions

Participants of the AO+MI_BT and MI_BT groups participated in a 4-week training regimen consisting of 16 non-physical training sessions (four sessions per week). All sessions lasted 30 min and were surveyed and supervised by the authors of the study. The duration and the number of training sessions was chosen based on previous studies investigating the effect of physical balance training (Taube et al., 2007a; Schubert et al., 2008). Participants of the CON group maintained their normal physical activities throughout the experimental period and were measured again 4 weeks after their initial test. The CON group was measured to exclude (short-term) learning effects potentially obtained during the initial testing session.

#### Observational balance training

During AO+MI_BT, participants were sitting in a darkened room in front of a computer screen. Video clips showed a person filmed from behind performing different balance tasks with the left leg and with the right leg. For each exercise, left and right sides were displayed for 30 s each with 15 s of rest in between. There was a 30-s rest between different exercises that involved balancing on air cushions (dynair), soft mats (airex), large ankle disks (custom made), free-swinging platforms (custom made), small ankle disks (custom made), and custom-made balance boards (for illustration of the devices see Gruber et al., 2007a). The difficulty of the exercises was progressively increased from week 1 to 4. In week 2, participants in the videos had to balance and at the same time guide a ball behind their back and through their legs. In week 3, external “perturbations” were shown: participants had to catch and pass balls while balancing. In week 4, participants were shown while balancing with their eyes closed, leading to the most pronounced postural sway.

Throughout each training session, participants were encouraged to imagine that they were actually the person in the video and to feel the sensation of postural sway and balancing, as this kind of mental involvement was shown to induce greater changes in corticospinal excitability (Roosink and Zijdewind, 2010) and larger brain activity in pre-motor cortices, SMA, primary motor cortex, basal ganglia, and cerebellum than passive observation (Taube et al., 2015). Furthermore, they were asked to watch the videos with intense concentration and a close focus on the task at hand. Participants observed for each leg two series of exercises performed on the different devices.

#### Motor imagery balance training

The MI_BT also lasted for 30 min and started with a short relaxation protocol (Hickman et al., 1977) in order to focus concentration on the participant's own body. Afterwards, participants were asked to imagine their bodies in the first perspective (kinesthetic motor imagery) so that they concentrated not only on performing specific postural tasks but also on feeling the sensations arising from doing these tasks (Grangeon et al., 2011).

For two main reasons we asked participants not only to imagine the exercises that the AO+MI_BT group saw on video but to add many different postural tasks. First, participants were not familiar with balance training devices—neither with their shape and appearance nor with the feeling of exercising on them. Thus, imagining these tasks would have been difficult if not impossible for most of the participants. Furthermore, recent studies indicate that physical experience of a task is an important pre-requisite to activate relevant motor representations in the brain (Olsson et al., 2008; Olsson and Nyberg, 2010, 2011). Second, from a motivational point of view, imagining the same tasks over and over again for 4 weeks would have been problematic. Thus, we conceptualized a training that may well be applied in a practical setting such as during an immobilization period. Participants were asked to imagine postural tasks they knew for the most part from everyday life such as balancing on one leg, keeping balance on a boat sailing through a stormy sea, jumping from stone to stone in a river bed, balancing on a narrow beam, and so forth. In addition, participants were asked to imagine keeping balance on the devices that were shown to the participants of the AO+MI_BT group. Throughout each training session, participants were frequently encouraged to imagine the task vividly so that they could feel the sensations arising from each balance exercise.

### General experimental procedure

The experimental procedure was the same in the pre- and post-measurements. The post-measurement occurred approximately 48 h after the last training session in order to minimize the influence of “mental” fatigue. Participants were barefoot during all measurements. The order of conditions was randomized to avoid sequence of order effects. First, H-reflex recruitment curves were recorded during bipedal stance and unipedal stance (see “Peripheral nerve stimulation”). Second, postural stability was assessed on a free-swinging platform (Posturomed™) with and without perturbation and by means of a functional reach test (see “Balance tests”). Third, the maximal rate of force development (RFD_max_) was recorded during isometric plantarflexions (see “Explosive strength”). Finally, participants were asked to perform maximal squat jumps and countermovement jumps (see “Jump tests”).

### Balance tests

Postural stability was assessed with different tests. First, postural control was evaluated on a balance device (Posturomed) that allowed platform sway in the transversal plane (for technical details see Mueller et al., 2004) and was shown to have good test-retest reliability (Boeer et al., 2010). For this purpose, participants stood with one leg on the free-swinging device and were asked to sway as little as possible during the measurement period of 15 s. A period of 15 s was chosen due to the fact that longer exposures than 15 s may lead to fatigue, especially in participants that struggle to keep their balance on this device. Anterior-posterior and medio-lateral sway paths were recorded by joystick potentiometers connected to the moveable platform. To minimize the influence of short-term learning effects, subjects were given 2 min on the device to familiarize with the task (in accordance with Keller et al., 2012). Afterwards, the cumulative sway paths of three trials were averaged for each condition and values obtained before and after training were compared.

In the second test, participants were standing with both legs on the Posturomed. However, this time a medio-lateral perturbation was applied. For this purpose, the device was moved out of the neutral position and was magnetically fixed with a displacement of 2.5 cm. After a random time in this stable position, the experimenter released the magnet and the platform started to swing. Thus, subjects could not anticipate the perturbation. Participants were asked to reduce the oscillations as fast and as thoroughly as possible within the first 15 s. Before the measurements started, each subject was exposed to 5 perturbations in order to familiarize with the task and thus, to reduce the influence of short-term learning effects (in accordance with Keller et al., 2012). Afterwards, three trials were recorded and averaged.

The third balance test involved a classical functional reach test in which participants had to lean forward as far as possible (Duncan et al., 1990). The maximum forward lean was measured during three trials using a custom-built device consisting of a slider with very low resistance that had to be slowly pushed forward with the arm.

### Explosive strength

The maximal rate of force development (RFD_max_ [dF/dt]) was determined during isometric plantarflexions (in line with previous studies evaluating RFD after physical BT, e.g., Gruber et al., 2007a). Participants were seated with hip, knee and ankle angles at 90° and feet placed on a force transducer (AMTI MC3A-500, Watertown, MA, USA). A custom-built device guaranteed isometric contractions by placing a rigid strap around the thigh above the knees. Then, participants were asked to generate maximal force within a minimal time by performing explosive isometric plantarflexions. After participants were accustomed to the task, they performed three maximal trials and the mean value of these trials was used for further analysis.

### Jump tests

Participants were tested in two jump conditions: squat jumps (SJs) and countermovement jumps (CMJs). All jumps were performed on a force plate (AMTI OR6-7, Watertown, MA, USA) with maximum effort. Jump heights of three maximal squat and three maximal countermovement jumps were calculated based on the formula: jump height $=\frac{{\text{g*t}}^{2}}{8}$, where t is the duration of the flight phase and g represents the acceleration of gravity. For all jumps, participants retained their hands akimbo to avoid supportive movements of the arms during jumping. The mean of three trials was calculated for each jump condition.

### Electromyography

Muscular activity was recorded during all balance tasks on the posturomed by means of bipolar surface electromyography (EMG) in line with the SENIAM guidelines (Hermens et al., 2000). After skin preparation, electrodes (Blue Sensor P, Ambu A/S®, Bad Nauheim, Germany) were firmly attached to the skin in line with the direction of the underlying muscle fibers of m. soleus (SOL), medial m. gastrocnemius (GAS), m. tibialis anterior (TA) and m. peroneus longus (PER) of the right leg. EMG signals were sampled at 4 kHz, amplified (x1000) and band-pass filtered (10–1000 Hz).

### Peripheral nerve stimulation

With an inter-stimulus interval of 4 s, rectangular current pulses of 1 ms each were delivered to the posterior tibial nerve by a constant current stimulator (AS100 Alea Solutions®, Switzerland). The anode, a 5 × 5 cm dispersal pad, was fixed on the anterior aspect of the knee just below the patella. The cathode (2 cm in diameter) was placed in the popliteal fossa and moved stepwise until the best position for eliciting an H-reflex in the soleus muscle was found. The best position was defined as the spot where the largest H-reflex could be elicited without eliciting responses in the TA muscle. The cathode was fixed with rigid tape. First, H-reflex recruitment curves were recorded during normal upright stance and the stimulation intensity was increased until the maximal M-wave (M_max_) was AO+MI_BTained. When the M-wave ceased to increase and a plateau was reached, the stimulation intensity was further markedly increased in order to ensure that M_max_ was indeed AO+MI_BTained. The maximal H-reflex was subsequently expressed relative to M_max_ (H_max_/M_max_ ratio). The identical procedure was applied during upright bipedal stance and one-legged stance, and while balancing on the Posturomed.

### Data analysis and statistics

The sway path of the Posturomed was summed up during 15 s over the course of each of the three trials and subsequently averaged without perturbation and again over the course of each of the three trials with perturbation. A repeated measures ANOVA with the within-subject factors TIME (pre, post) and CONDITION (one-legged stance, perturbation task) and the between-subject factor GROUP (MI_BT, AO+MI_BT, CON) was calculated [2 (TIME) × 2 (CONDITION) × 3 (GROUP)]. For the functional reach test, an ANOVA with the factors TIME (pre, post) and GROUP (MI_BT, AO+MI_BT, CON) was calculated taking into account the reach distances measured in cm in pre- and post-measurements.

EMG activity was analyzed as root mean square values (RMS) during the entire 15 s of balancing on the Posturomed for both unperturbed stance on one leg and bipedal stance with perturbation. A repeated measures ANOVA with the factors TIME (pre, post), CONDITION (no perturbation vs. perturbation), MUSCLE (SOL, GAS, TA, PER) and GROUP (MI_BT, AO+MI_BT, CON) was calculated. H_max_/M_max_ ratios were calculated based on the maximal H-reflex and M-wave amplitudes of the H-reflex recruitment curves and subsequently analyzed by a repeated measures ANOVA with the within-subject factors TIME (pre, post) and CONDITION (bipedal stance, one-legged stance, bipedal stance on the Posturomed) and the between-subject factor GROUP (MI_BT, AO+MI_BT, CON).

The effect of training on the explosive strength (RFD_max_) was analyzed by means of a repeated measures ANOVA with the factors TIME (pre, post) and GROUP (MI_BT, AO+MI_BT, CON). For both SJs and CMJs, the jumps with maximal height were selected for each participant and compared before and after training using a repeated measures ANOVA with the factors TIME (pre, post), and GROUP (MI_BT, AO+MI_BT, CON). In case of significant *F*-values (*P* < 0.05), *post-hoc* pairwise comparisons with Bonferroni-Holm corrections were conducted and the corrected values are displayed throughout the manuscript. SPSS 20 software was used for all statistical analysis. Data are presented as group mean values ± standard deviation, if not indicated differently.

## Results

### Balance tests

#### Posturomed

**Figure 1:**
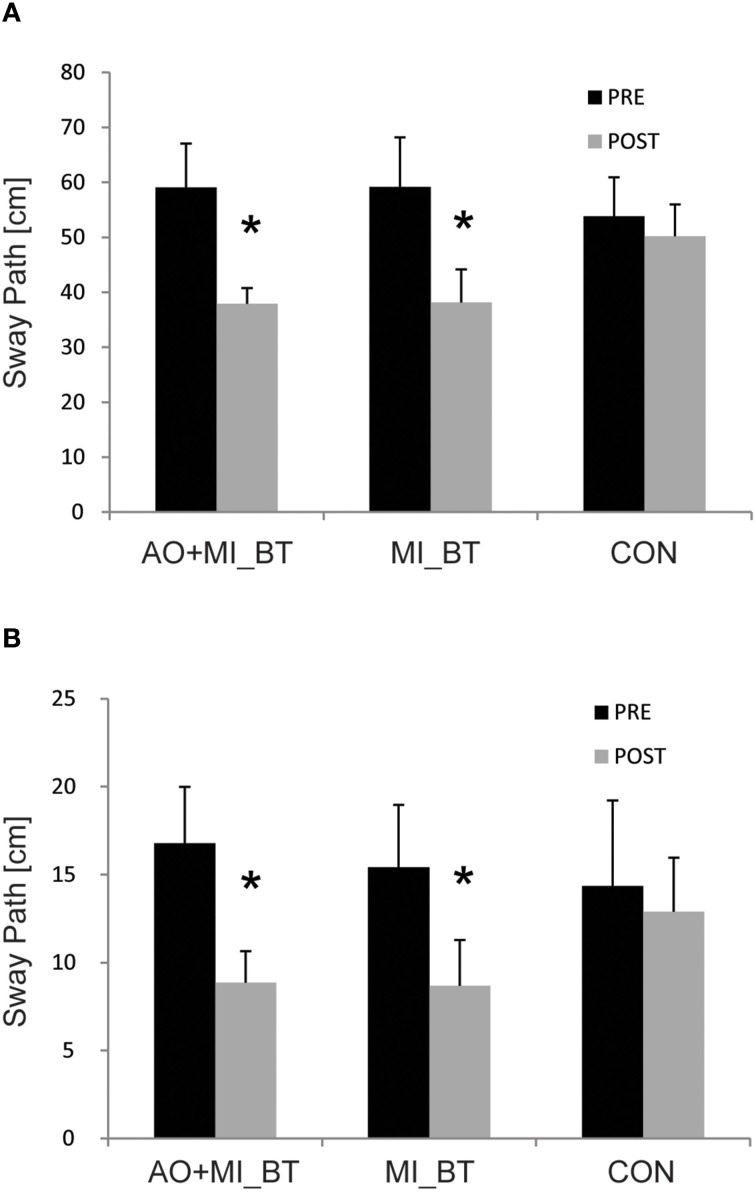
**Balance performance before and after training**. Participants displayed a significantly reduced sway path when standing on a free-swinging platform with **(A)** and without perturbation **(B)** after participating in motor imagery (MI_BT) or observational balance training (AO+MI_BT). The sway path of the control group (CON) did not change. Data are presented as group mean and stars (*) indicate significant suppression of the mean sway paths (* < 0.05).

**Figure 2:**
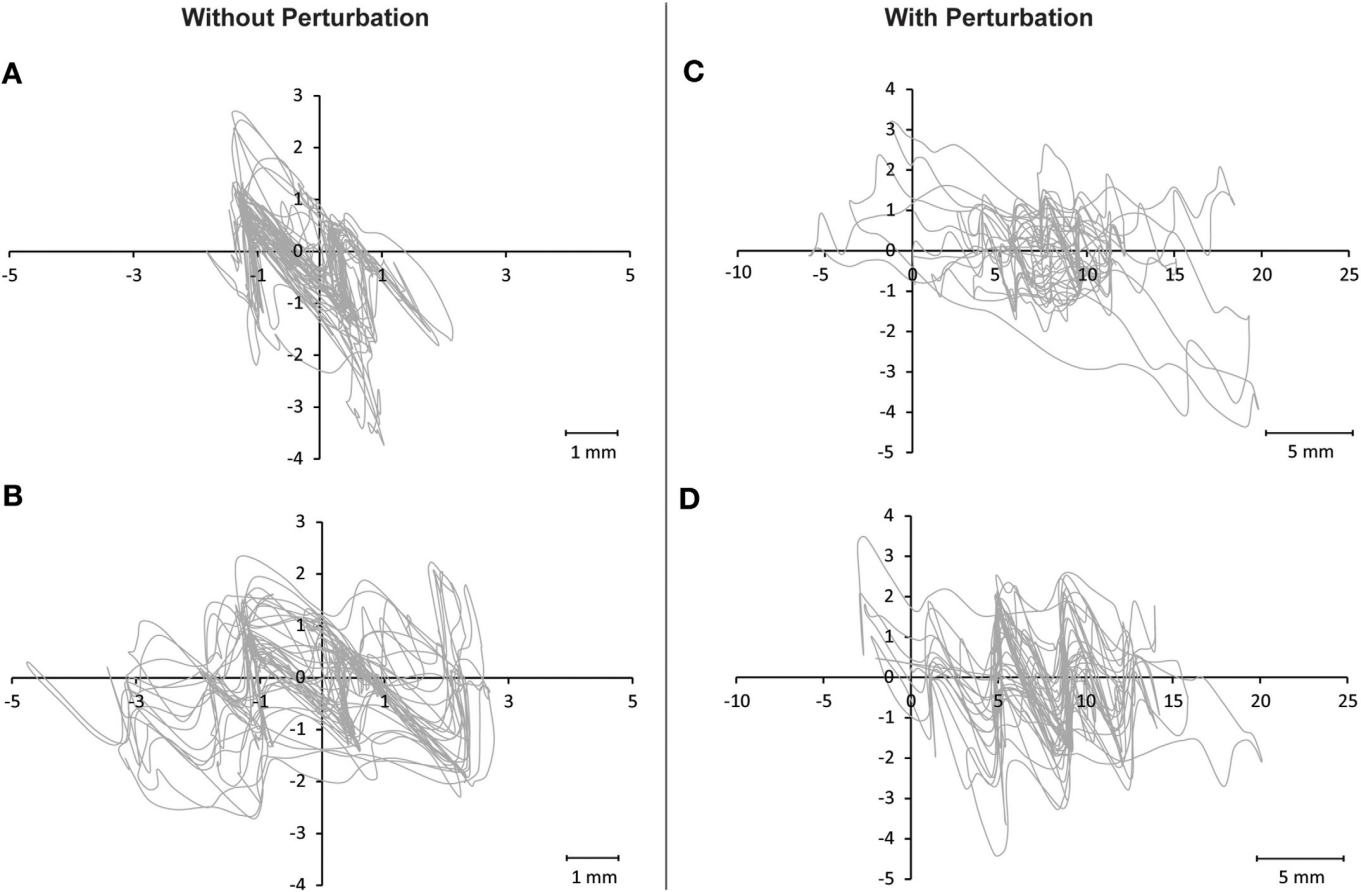
**Illustration of sway paths on the Posturomed**. Two arbitrary chosen trials of one participant are displayed for the condition involving no perturbation **(A,B)** and for the condition with perturbation **(C,D)**. It can be seen that although the length of the sway path was comparable for **(A,B)** and for **(C,D)**, respectively, each trial nevertheless displays a distinct sway pattern that was different from trial to trial.

Comparison of balance performance before and after training revealed a significant TIME [*F*_(1, 32)_ = 24.56; *P* < 0.001] and TIME × GROUP effect [*F*_(2, 32)_ = 3.56; *P* = 0.04; Figure 1]. *Post-hoc* analysis indicated that the sway path was significantly reduced in the MI_BT group in the perturbation condition (*P* = 0.024) and for the single-leg stance on the Posturomed (*P* = 0.049). *Post-hoc* tests for the AO+MI_BT group demonstrated also significantly improved performance for both the one-legged stance without perturbation (*P* = 0.004) and the bipedal stance with perturbation (*P* = 0.044). Interestingly, the sway pattern was distinct from trial to trial for both unperturbed (Figures 2A,B) and perturbed stances (Figures 2C,D).

#### Functional reach test

Analysis revealed a significant TIME effect [*F*_(1, 32)_ = 4.83; *P* = 0.035] but no TIME × GROUP effect [*F*_(2, 32)_ = 0.23; *P* = 0.794]. This was due to the fact that not only the MI_BT (+1 ± 2.4 cm) and the AO+MI_BT groups (+0.6 ± 2.1 cm) but also the CON group (+0.5 ± 1.3 cm) showed improvements.

### Explosive strength

**Figure 3:**
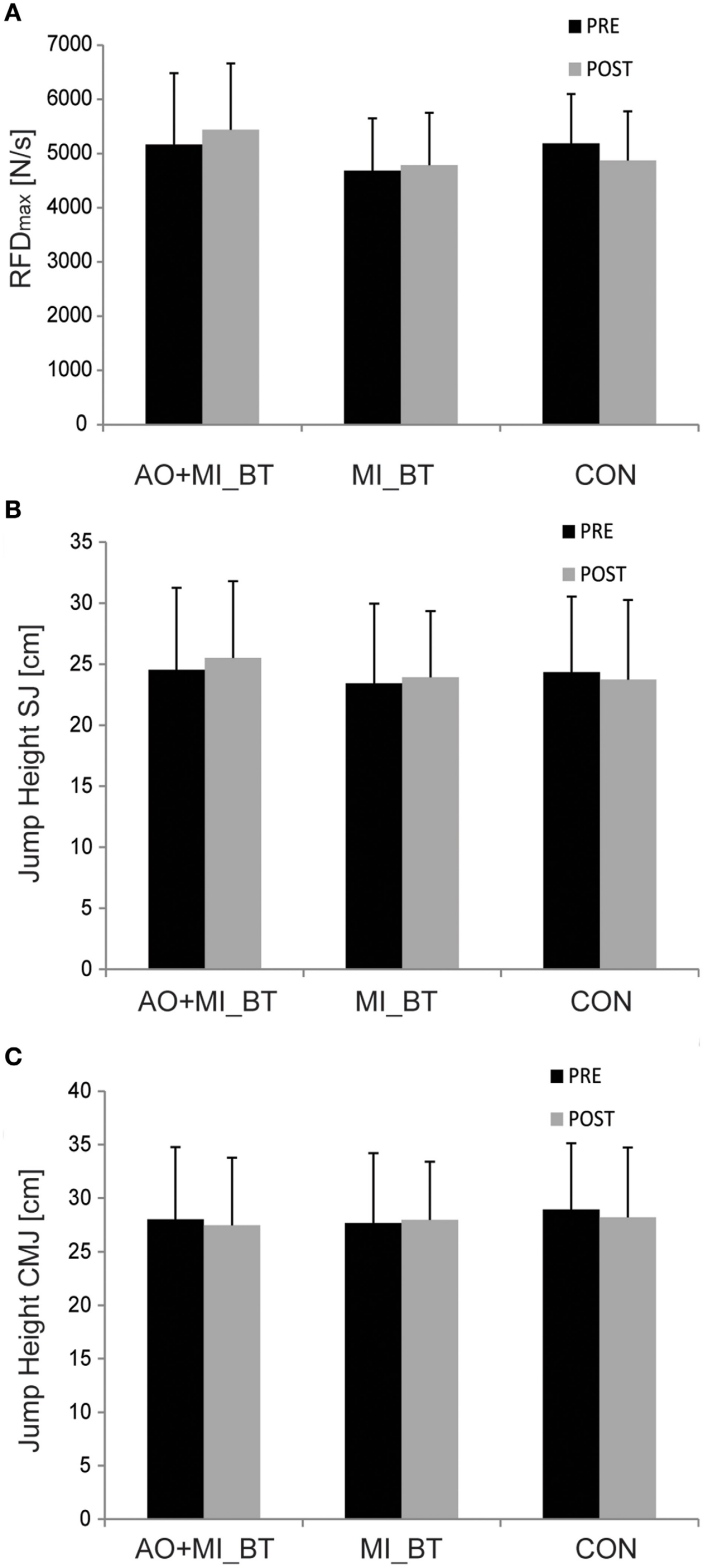
**Explosive force and jump behavior before and after training**. Neither the rate of force development (RFD; **A**) nor the jump height of squat (SJ; **B**) and countermovement jumps (CMJ; **C**) changed after motor imagery (MI_BT) or observational balance training (AO+MI_BT). Data are presented as group mean.

The explosive strength (RFD_max_) remained unchanged after training [TIME: *F*_(1, 31)_ = 0.013; *P* = 0.911] and there was no TIME × GROUP interaction [*F*_(2, 31)_ = 1.002; *P* = 0.379; Figure 3A].

### Jump tests

Jump heights did not change in the post-measurement [TIME: *F*_(1, 30)_ = 0.248; *P* = 0.622] and were not different between groups over time [TIME × GROUP: *F*_(2, 30)_ = 1.521; *P* = 0.235; Figures 3B,C].

### Muscular activity

Muscular activity did not change after the training [TIME: *F*_(1, 31)_ = 0.054; *P* = 0.818] and was not different between groups [TIME × GROUP: *F*_(2, 31)_ = 0.071; *P* = 0.932]. There was also no TIME × MUSCLE [*F*_(3, 93)_ = 0.598; *P* = 0.618] or TIME × MUSCLE × GROUP interaction [*F*_(6, 93)_ = 0.275; *P* = 0.947]. We only found a CONDITION effect (perturbation vs. no perturbation), indicating that muscular activity was higher in the perturbation task [*F*_(1, 31)_ = 66.634; *P* < 0.001], but again, no changes over time were noticeable [TIME × CONDITION: *F*_(1, 31)_ = 0.784; *P* = 0.383; results are not displayed due to space limitations].

### Peripheral nerve stimulation

**Figure 4:**
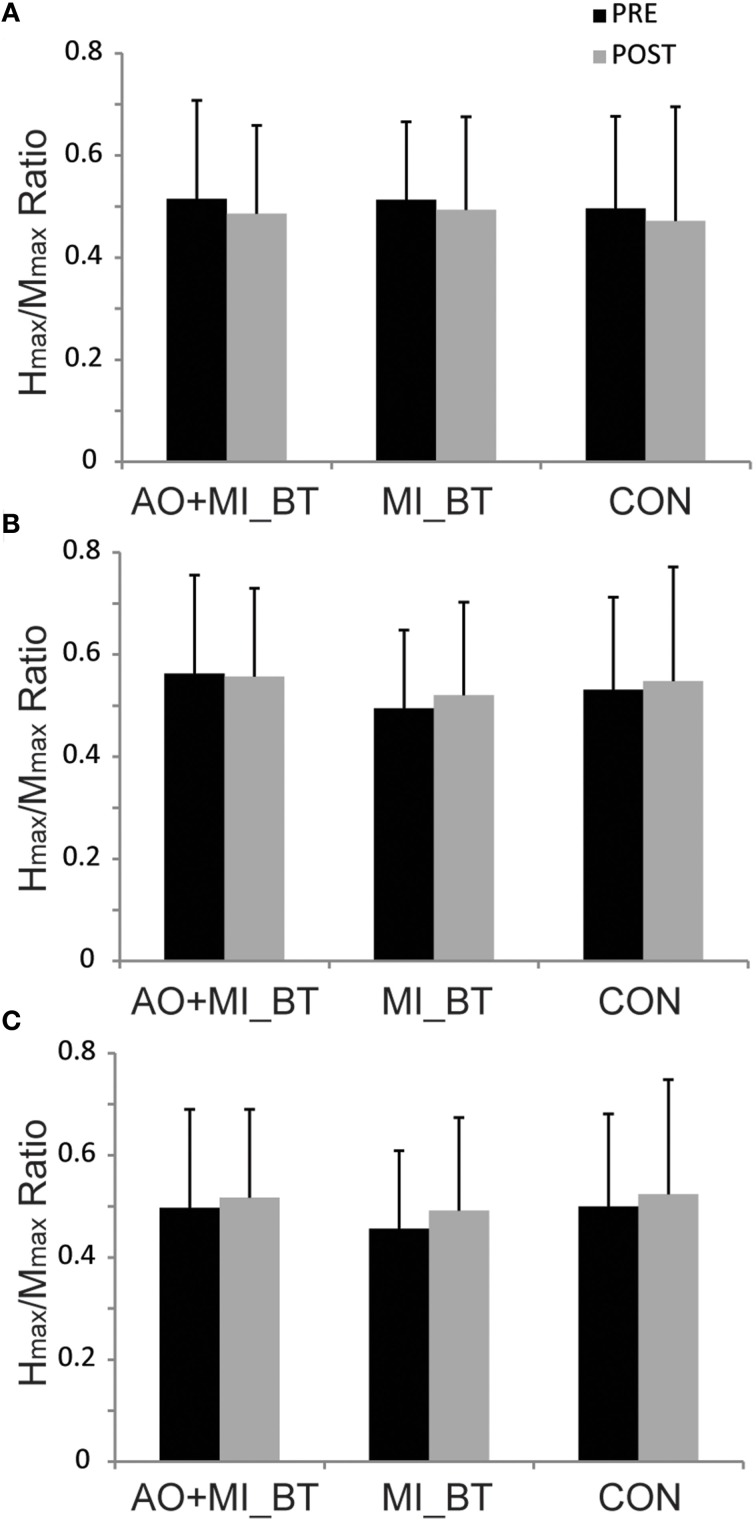
**H_max_/M_max_ ratios before and after training**. The maximal H-reflex (H_max_) was expressed with respect to the corresponding maximal M-wave (M_max_) in three different postural tasks: **(A)** upright bipedal stance, **(B)** one-legged stance, and **(C)** bipedal stance on the Posturomed. The H_max_/M_max_ ratios did not change in any of these conditions after motor imagery balance training (MI_BT), or observational balance training (AO+MI_BT), or in the control group (CON). Data are presented as group mean.

H_max_/M_max_ ratios remained unchanged in the post-measurement [TIME: *F*_(1, 29)_ = 0.005; *P* = 0.944] and were not different between groups [TIME × GROUP: *F*_(2, 29)_ = 0.025; *P* = 0.976; Figure 4].

## Discussion

The current data provide for the first time evidence that mental as well as observational training can improve performance in an unstable and thus non-predictable environment. Specifically, postural control was improved on an unstable support surface both with and without external perturbation. In contrast to the “transfer adaptations” known from physical BT such as increased explosive strength (Gruber et al., 2007a) and increased jumping performance (Taube et al., 2007b), no such adaptations were observed after non-physical mental BT. Furthermore, the well-documented reduction in spinal H-reflex excitability after physical BT (Trimble and Koceja, 1994; Taube et al., 2007a; Gruber et al., 2007b; Keller et al., 2012) was not apparent after MI_BT and AO+MI_BT. Thus, it can be concluded that mental, non-physical BT induces adaptations similar to those induced by physical BT in some respects but also displays fundamental differences in other aspects.

### Static and dynamic balance performance after MI_BT and AO+MI_BT

Many studies indicate that motor imagery (Hallett et al., 1994; Sirigu et al., 1995; Stephan et al., 1995; Lotze et al., 1999; Gerardin et al., 2000; Grezes and Decety, 2001; Jeannerod, 2001; Kimberley et al., 2006) as well as observation of movements (Gallese et al., 1996; Grezes and Decety, 2001; Neuper et al., 2005) activate brain regions that are also active during actual task performance. Based on these findings, it is assumed that activation of (motor) representations is also responsible for behavioral adaptations after non-physical training. For instance, motor imagery was shown to improve the acquisition of motor sequence learning paradigms (e.g., Debarnot et al., 2011a), tracking tasks (Debarnot et al., 2011b), strength exercises (e.g., studies summarized in table 1 in Reiser et al., 2011), and a variety of sport activities (e.g., Guillot et al., 2013). Similarly, observational learning was reported to promote acquisition of movement sequences (van der Helden et al., 2010), adaptations to force fields (Mattar and Gribble, 2005), the learning of guitar chords (Higuchi et al., 2012), increasing strength (Porro et al., 2007) and an improvement in sports performance (e.g., Lawrence et al., 2013).

As mentioned above, motor imagery (Hamel and Lajoie, 2005) as well as observational training (Tia et al., 2010) enhanced performance in postural tasks. However, neither the last two studies nor all other previously introduced motor imagery and observational approaches have ever tested whether non-physical training can improve balance performance in an unstable and unpredictable environment. Counteracting perturbations in an unstable environment relies heavily on integration of sensory feedback to select adequate movement trajectories. The results of the present study are therefore remarkable, as they demonstrate for the first time that different forms of non-physical training (MI_BT and AO+MI_BT) not only promote motor learning of “rigid” tasks but also improve performance of highly variable and non-predictable motor actions. Importantly, the participants were not mentally trained for the specific tasks on the Posturomed and these tasks can therefore be considered as postural transfer tasks (for more details see “Transfer adaptations”). Thus, the acquisition of balance skills seems not to be restricted to the specific movements that were performed during the non-physical training sessions but to extend to postural movements that are similar.

### Movement observation vs. motor imagery learning

In a recent study, Gatti et al. (2013) compared the effectiveness of action observation and motor imagery when learning a complex motor task. Participants of the observational group outperformed the motor imagery group so that the authors concluded that movement observation might be better suited to learning a new motor task, “at least in the fast early phase of motor learning” (Gatti et al., 2013). Although it is of great relevance to compare the effectiveness of motor imagery and observational learning, the conclusions of this study may have been pre-mature, as the design of the study did not allow the assessment of the baseline level before training. Thus, potential differences in performance before the training cannot be excluded. Furthermore, the authors acknowledge that the motor imagery group had more difficulties in AO+MI_BTaining an appropriate understanding of the action when no visual cues about the task were presented. Finally, the design did not allow retention tests. It can therefore not be excluded that the motor imagery group caught up later on.

Unfortunately, the present study also does not allow a direct comparison of the effects of action observation and motor imagery. We chose two different protocols for the MI_BT and the AO+MI_BT groups (see the section on “Materials and Methods” for further details). Thus, participants of the two groups did not imagine/observe the same movements for the same amount of time. This is certainly a limitation of the current study but at the same time was probably necessary in order to obtain significant adaptations for the motor imagery group. Recent studies indicate that previous physical experience is necessary to imagine a motor task properly and to activate the corresponding motor representations in the brain (Olsson et al., 2008; Olsson and Nyberg, 2010, 2011). Thus, motor imagery seems to be limited to tasks that have been physically executed previously. To some extent, this might also be true for observation of motor tasks, as several studies have demonstrated different brain activation patterns in familiar actions than in less familiar movements (e.g., Calvo-Merino et al., 2005, 2006). However, it is also known from observational learning studies that participants are able to acquire not only high-level information about the form of a new movement such as learning a finger-tapping sequence (e.g., Kelly et al., 2003) but may also learn novel patterns of generating muscle forces in an unknown force environment (Mattar and Gribble, 2005). Thus, participants are able to learn “what” movements should be done (e.g., sequence task) and—even more importantly—“how” these movements have to be done (e.g., application of the correct force) by simply observing the motor actions of others. Consequently, observational learning does not seem to require prior experience of the task in the same way as learning by motor imagery does. There are therefore differences in how motor imagery and movement observation promote learning that are also evident when regarding brain activation patterns (e.g., Neuper et al., 2005) and responses to short-term immobilization (Bassolino et al., 2014). Nevertheless, the current data display similar improvements in postural control after MI_BT and AO+MI_BT so that it may be speculated that a combination of both would have been most effective.

### Transfer adaptations

It was shown that mental strength training of the left little finger increased strength not only for the left but also for the right little finger (Yue and Cole, 1992). Thus, non-physical training may cause interlimb transfer similar to the effects reported after physical training. However, little is known about the ability to transfer certain knowledge or capacities that were acquired during non-physical (mental) learning of one specific task to a second, in fact similar but nevertheless distinct, task. The results of the present study therefore demonstrate for the first time that MI_BT and AO+MI_BT improve performance of highly variable and non-predictable postural exercises that were not specifically trained. This means that participants did neither mentally rehearse nor observe the specific tasks on the Posturomed during training but nevertheless improved their performance on this device. Furthermore, when regarding the exemplary sway paths in Figure 2 it becomes obvious that each trial shows a distinct and unique pattern. Thus, motor imagery and observational learning of postural tasks are effective to variably counteract dynamic perturbations, which underline the functional significance of the present findings (please see “*Functional significance of the current results*”). However, while MI_BT and AO+MI_BT revealed adaptations similar to those produced by physical balance training with respect to balance performance, no transfer adaptations in RFD_max_ or the jump performance were detected although such transfer adaptations occur after physical balance training (Gruber and Gollhofer, 2004; Gruber et al., 2007a; Taube et al., 2007b; Boccolini et al., 2013). This discrepancy may result from the fact that non-physical training does not promote integration and processing of afferent feedback. Muscle afferent feedback provides an important facilitatory influence on human α-motoneurons (Macefield et al., 1993) and it was proposed that physical balance training improves integration of afferent feedback (Gruber and Gollhofer, 2004) so that this could explain improvements in the RFD_max_ after conventional but not after mental training.

### Spinal excitability

Reduction in spinal excitability indicated by reduced H-reflex responses is a well-known adaptation in response to physical balance training (for review Taube et al., 2008). It is assumed that suppression of Ia-afferent transmission inhibits unwanted joint oscillations that originate from muscle stretch reflexes (for review Taube et al., 2008; Keller et al., 2012). Based on the observation that the H-reflex was modulated during motor imagery of motor actions (Oishi et al., 1994; Hale et al., 2003) and after 10 weeks of mental up- or down-training of the H-reflex (Thompson et al., 2009), it was hypothesized that mental balance training may also alter spinal reflex excitability. However, this was not the case in the current study. Thus, it seems unlikely that altered processing of spinal reflexes influenced performance outcomes after MI_BT or AO+MI_BT. In this way our study supports previous findings showing that improved postural control is not necessarily associated with a reduction of the soleus H-reflex (Beck et al., 2007; Schubert et al., 2008). Consequently, future studies should evaluate supraspinal adaptations especially as a close interrelation of supraspinal plasticity and behavioral changes in postural control (Taube et al., 2007a; Taubert et al., 2010, 2011) has been observed.

### Functional significance of the current results

Although the high plasticity of the human central nervous system has to be considered as advantageous under normal conditions in order to learn new skills, adapt to unfamiliar environments and compensate sensorimotor disorders, it may comprise a risk when the body is inactive due to health issues (e.g., pain, lesions, tendon ruptures or broken bones) or environmental limitations (e.g., space flight). Merzenich et al. (1983) were the first, followed by others (Allard et al., 1991; Benedetti, 1991; Brasil-Neto et al., 1992; Merzenich and Jenkins, 1993), to show that inactivity of a body part reduces the representation of this part in the somatosensory cortex. Recent studies indicate that even relatively short (5- to 8-week) periods of disuse affect this internal representation (de Jong et al., 2003; Zanette et al., 2004). Interestingly, disuse is also associated with an impaired ability to perform motor imagery of the affected limb (Fiorio et al., 2006). The close interrelation of actual and mental execution of movement can also be seen during rehabilitation as subjects who performed imagined movements of their immobilized limb displayed less physiological and behavioral impairments (Mulder, 2007; Malouin and Richards, 2010). However, little is known about prevention of postural decline by means of non-physical practice. Hamel and Lajoie (2005) demonstrated that motor imagery can reduce postural sway in static bipedal stance. The present study adds an important new aspect by showing improved performance in dynamic perturbation tasks after non-physical training. Previously it was demonstrated that simple balance tests like bipedal stance on a solid base of support can be non-adequate means when testing for functional relevant improvements in postural control (Taube et al., 2010) and unreliable predictors for the risk of falling (Horak et al., 1992). Studies investigating the effect of Parkinson disease on human balance support this assumption. For example, Smithson et al. (1998) did not find any impairments in postural control when patients were tested in simple static balance tasks. The same authors, however, observed significant constraints of postural stability when the same patients were tested in more challenging postural tasks. Other studies indicate that parkinsonian patients can even show “better” postural control than healthy controls when tested in static balance tests but displayed serious constraints when the demands of the postural tasks were increased (Horak et al., 1992; Schieppati et al., 1994). Thus, MI_BT and AO+MI_BT of dynamic balance tasks—or a combination of both—should be seriously considered during periods of immobilization in order to improve/maintain dynamic postural control.

## Acknowledgment

This work was supported by the Swiss National Science Foundation (SNF research grant 320030_144016/1).

### Conflict of interest statement

The authors declare that the research was conducted in the absence of any commercial or financial relationships that could be construed as a potential conflict of interest.
